# Supplementary material for: Taxonomic Revision of Genus Ephedra Tourn. ex L. in Egypt with Intra-Gender Diversity in Morphometric Traits and Fatty Acid Composition
Source: Plants (Basel). 2024 Sep 1;13(17):2442. doi: 10.3390/plants13172442 (PMC11397020; doi:10.3390/plants13172442)
Supplement: Supplementary file 1 [file plants-13-02442-s001.zip › plants-3138724-supplementary.pdf]

Table S1: Illustrates the morphological characteristics studied of the male and female species of *Ephedra*.

FORM / CHARACTER	*E. alata* female	*E. aphylla* female	*E. ciliate* female	*E. foemina* female	*E. pachyclada* female
**Plant form**	Shrub	Shrub	Shrub	Shrub	Small shrub
**Shrub**					
**Habitat (wadi/mountain)**	Wadi	Wadi	Wadi-mountain slopes	Mountain slopes-rocky wadi	Wadi-mountain slopes
**Plant height**	Up to 1 m	Up to 1.5 m	Up to 3 m	Up to 4 m	Up to 40 cm
**Growth form**	Erect	Climbing	Climbing	Climbing and prostrate	Erect
**Twigs (branches)**					
**Origin**	Opposite	Whorled	Pseudo whorled-opposite	Opposite-whorled	Opposite
**Color**	Yellowish-green	Blue-green	Blue-green to grey-green	Dark green	Whitish-green
**Structure**	Stout branches	Long, tortuous branches	Striated, flexuous and narrow branches	Thin, wiry branches	Stiff branches and thickened upright
**Shape**	Rounded	Rounded	Rounded	Rounded	Rounded
**Texture**	Scabrous	Scabrous	Hairy	Glabrous	Glabrous
**Stem**					
**Number of stems**	Moderate branched	Moderate branched	Moderate branched	Branched	Much branched
**Origin of branches**	Branched at base	Branched at base	Branched at base	Branched allover	Much branched at nodes
**Number of lateral branches at a node**	3-5	2-5	2-5	2-4	10-13
**Size of internode L x W mm**	37-51 × 1.5-2 mm	30-45 × 1-2.9 mm	20-69 × 1-2 mm	20-37 × 1-2 mm	10-25 × 1-2 mm
**Width of node mm**	2-3 mm	2-4 mm	1-4 mm	2-3 mm	2.5-3 mm
**Indumentum**	Papillose	Papillose-ciliate	Ciliate	Papillose	Smooth-minutely papillose
**Trichomes type**	-	Unicellular-uniseriate	Unicellular-uniseriate	-	-
**Stem grooving**	Clear-moderate grooves	Moderate-clear grooves	Non-conspicuous grooves	Non-conspicuous grooves	Non-conspicuous grooves

Leaf					
**Shape**	Elliptical	Triangular	Filiform	Elliptical	Elliptical
**Size of leaf L x W mm**	1.8-3 × 0.8-1.5 mm	2-4 × 0.4-0.8 mm	5-15 × 0.1-0.5 mm	0.8-2 × 0.5-1.7 mm	1.8-2.5 × 1-1.5 mm
**Length of united part**	1.5-3 mm	0.5-2 mm	0.5-2 mm	0.5-2 mm	1.8-2 mm
**Length of free part**	0.2-0.5 mm	1-2.5 mm	4.5-13 mm	0.2-0.3 mm	0-0.2 mm
**Length of leaf sheath**	1.5-3 mm	0.9-1 mm	0.2-1 mm	0.5-2 mm	1.8-2 mm
**Leaf arrangement**	Opposite	Opposite-whorled	Whorled-opposite	Opposite-whorled	Opposite
**Number of whorls of leaves**	2	2-(3)	2-3	2-(3)	2
**Presence of trichomes on leaf margin**	Present	Present	Present	Absent	Absent
**Trichomes density on the leaf margin**	Sparsely	Densely	Densely	-	-
**Trichomes type on the leaf margin**	Unicellular-uniseriate	Unicellular-uniseriate	Unicellular-uniseriate	-	-
**The presence of trichomes on the sheath margin**	Present	Present	Present	Absent	Absent
**Trichomes density on sheath margin**	Rare	Sparse	Moderate	-	-
**Trichomes type on the sheath margin**	Unicellular-uniseriate	Unicellular-uniseriate	Unicellular-uniseriate	-	-
**Cone bract**					
**Cone bract size Lx W mm**	2.5-3.5 × 1.5-2 mm	1-1.5 × 0.5-1 mm	1.5-3 × 0.5-2 mm	1-1.5 × 1-1.5 mm	0.8-1.5 × 1 mm
**Number of strobili /cone**	1	1	1	1	1
**Number of pairs of bracts/ cone**	4-5-(6)	2-3	2-3	3-4-(5)	2-3
**Number of cones at each node**	5-6	3	3-4	3-4	3-4
**Bract connection**	Free	Fused	Fused	Fused	Fused
**Shape**	Elliptical	Ovate	Ovate	Elliptical	Ovate
**Apex**	Obtuse-notched	Obtuse	Acute-obtuse	Obtuse	Acute

**Margin**	Entire	Ciliate	Ciliate	Entire	Entire
**Length of cone bract: cone**	Less than 1/3	Less than 1/3	Less than 1/3	Less than 1/3	Less than 1/3
**The shape of two bracts (equal or sub-equal)**	Sub equal	Equal	Equal	Sub equal	Sub equal
**Shape of mid-region**	Have two longitudinal green stripes	In the middle, very dark line and the rest is transparent	Raphe (U- shape)	Raphe (U- shape)	Bract with transparent margin
**Trichomes outside**	Glabrous	Hairy	Hairy	Glabrous	Glabrous
**Trichomes density outside**	-	Densely	Densely	-	-
**Trichomes inside**	Hairy	Hairy	Hairy	Glabrous	Glabrous
**Trichomes density inside**	Sparsely (only 3-4 small hairs)	Densely	Densely	-	-
**Trichomes type**	Unicellular-uniseriate	Unicellular-uniseriate	Unicellular-uniseriate	-	-
**Female cone bract**					
**Number of pairs of inflorescence bracts/ cone**	4-5	2	2	2-3	2-3
**Base connection**	Free	Fused	Fused	Fused	Fused
**Outer bract of female strobilus**					
**Shape**	Orbicular	Oblong-elliptical	Ovate	Obovate	Oblong-ovoid
**Apex**	Rounded	Obtuse-rounded	Obtuse-acute	Obuse-acute	Rounded
**Margin shape**	Shortly lacerate	Entire	Entire	Entire	Entire
**Texture of outer cone surface**	Ciliate	Glabrous	Glabrous	Glabrous	Glabrous
**Texture of inner cone surface**	Ciliate	Sparse ciliate	Dense ciliate	Glabrous	Glabrous
**Number of bracts/ strobilus**	2	2	2	2	2
**Size of outer flower bract/cone**	4-5 × 2-3 mm	0.8-3 × 1-2.5 mm	2-3 × 1-2 mm	3-3.5 × 1-2 mm	3-5 × 2-3 mm
**The shape (equal or sub-equal)**	Equal	Equal	Equal	Sub equal	Equal

**Shape of mid-region**	Have two longitudinal green stripes	Raphe (U-shape) in the middle and transparent margin	Middle stripe and transparent margin	Raphe (U-shape) in the middle and transparent margin	The whole bract is dark and two stripes a little darker in the middle and transparent margin
**Trichomes inside**	Hairy	Hairy	Hairy	Glabrous	Glabrous
**Trichomes density inside**	Rare	Sparsely	Dense	-	-
**Trichomes type**	Unicellular-uniseriate	Unicellular-uniseriate	Unicellular-uniseriate	-	-
**Inner bract of female strobilus (seed bract)**					
**Shape**	Orbicular- circular	Elliptical	Elliptical	Elliptical	Elliptical- obovoid
**Apex**	Rounded	Obtuse-acute	Obtuse	Obtuse	Acute-rounded
**Margin**	Wavy, lacerate	Entire	Entire	Entire	Entire
**Size of inner strobilus bract/cone (Lx W mm)**	6 -7.5 × 3.5-4 mm	1.5-5.5 × 1-3 mm	4.5-7 × 1.5-3 mm	5-7 × 2-3 mm	4-6 × 2-3.5 mm
**The shape (equal or sub-equal)**	Sub equal	Sub equal	Sub equal	Equal	Sub equal
**Shape of mid-region**	Have two dark stripes in the middle and transparent margin	Have two dark stripes in the middle and transparent margin	The whole bract is dark it makes a raphe at the center transparent margin	Raphe (U-shape) in the middle and transparent margin	The whole bract is dark and transparent margin
**Trichomes outside**	Glabrous	Glabrous	Glabrous	Glabrous	Glabrous
**Trichomes inside**	Glabrous	Hairy	Hairy	Glabrous	Glabrous
**Trichomes density inside**	-	Rare	Dense	-	-
**Trichomes type**	-	Unicellular-uniseriate	Unicellular-uniseriate	-	-
**Origin of female cone clusters**	Dense axillary	Dense axillary	Terminal	Axillary	Axillary clusters
**Peduncle shape**	Straight	Curved	Straight	Curved	Short straight
**Length of united part of lower bracts**	1-1.5 mm	2-3.5 mm	2.5-6 mm	4-6 mm	1.5-2 mm
**No. of ovules per cone**	(1)-2	1-2	1-3	1-2	1-(2)
**Number of bract pairs in the female cone**	4-5	2-3	2	2-3	2-3

**Bract color of the mature cone**	Yellowish	Red	White-red	Red	Red
**Bracts of female cone (Free or fused)**	Free	Fused	Fused	Fused	Fused
**Length of innermost female strobilus bracts**	6-7.5 mm	1.5-5.5 mm	4.5-7 mm	5-7 mm	4-6 mm
**Fruit**					
**Texture of fruit (dry or fleshy)**	Dry	Fleshy	Fleshy	Fleshy	Fleshy
**Shape of fruit (berry or cone-like)**	Cone-like	Berry-like	Berry-like	Berry-like	Berry-like
**Shape of peduncle**	Erect	Erect	Flexible, curved	Flexible, curved	Flexible, curved
**Color of fruit**	Creamy	Red	White-red	Red	Red
**Seed**					
**Number of seeds/ strobilus**	(1)-2	1-2	1-3	1-2	1-(2)
**Length**	6.5-8 mm	5-6.5 mm	6-7 mm	6-8.5 mm	4-5.5 mm
**Width**	2.5-4 mm	2.5-4 mm	2.5-4 mm	1.8-2.5 mm	2.3-3 mm
**Size from front to back**	1-1.5 mm	2-2.5 mm	1.5-2 mm	0.3-0.5 mm	1-1.5 mm
**Color**	Brownish	Brownish-black	Brownish-black	Brownish	Brownish
**Shape**	Ovoid, tri-gone, and flattened	Ovate	Elliptical	Elliptical	Elliptical
**Apex**	Acuminate	Mucronate	Acute	Notched	Acute
**Base**	Rounded	Rounded	Rounded	Obtuse	Rounded
**Texture**	Scabrous	Smooth	Smooth	Scabrous	Scabrous
**The shape of the tubule" micropylar tubule"**	Straight	Straight	Straight	Straight	Curved
**Length of tubule**	2-3 mm	0.4-0.6 mm	0.5-0.8 mm	0.3-0.5 mm	1.5-3.5 mm

FORM / CHARACTER	***E. alata* male**	***E. aphylla* male**	***E. ciliate* male**	***E. foemina* male**	***E. pachyclada* male**
**Plant form**	Shrub	Shrub	Shrub	Shrub	Small shrub
**Shrub**					
**Habitat (wadi/mountain)**	Wadi	Wadi	Wadi-mountain slopes	Mountain slopes-rocky wadi	Wadi-mountain slopes
**Plant height**	Up to 1 m	Up to 1.5 m	Up to 3 m	Up to 4 m	Up to 40 cm
**Growth form**	Erect	Climbing	Climbing	Climbing and prostrate	Erect
**Twigs (branches)**					
**Origin**	Opposite	Whorled	Pseudo whorled-opposite	Opposite-Whorled	Opposite
**Color**	Yellowish-green	Blue-green	Blue-green to grey-green	Dark green	Whitish-green
**Structure**	Stout branches	Long, tortuous branches	Striated, flexuous and narrow branches	Thin, wiry branches	Stiff branches and thickened upright
**Shape**	Rounded	Rounded	Rounded	Rounded	Rounded
**Texture**	Scabrous	Scabrous	Hairy	Glabrous	Glabrous
**Stem**					
**Number of stems**	Branched	Moderate branched	Moderate branched	Branched	Much branched
**Origin of branches**	Branched at base	Branched at base	Branched at base	Branched allover	Much branched at nodes
**Number of lateral branches at a node**	2-4	3-6	3-5	2-4	10-14
**Size of internode L x W mm**	30-62 × 1.5-2 mm	32-68 × 1.5-3 mm	32-63 × 1-2.5 mm	13-27 × 1-2.5 mm	20-42 × 1.5-2 mm
**Width of node mm**	2.5-3.5 mm	2.5-3.5 mm	3-3.5 mm	2.5-3 mm	2-3 mm
**Indumentum**	Papillose	Papillose	Ciliate	Papillose	Smooth-minutely papillose
**Trichomes type**	Glabrous	Glabrous	Ciliate	Glabrous	Glabrous
**Stem grooving**	Moderate grooves	Non-conspicuous grooves	Non-conspicuous grooves	Non-conspicuous grooves	Non-conspicuous grooves
**Leaf**					
**Shape**	Elliptical	Triangular	Filiform	Elliptical	Elliptical

**Size of leaf L x W mm**	2-3.5 × 1-1.5 mm	1.5-3 × 0.5-1 mm	5-7 × 0.2-0.3 mm	1-2 × 0.5-1.5 mm	1.5-2× 1-1.5 mm
**Length of united part**	1-2.5 mm	0.5-1 mm	0.5-0.8 mm	0.5-2 mm	1.5-2 mm
**Length of free part**	1-1.5 mm	0.8-2 mm	2.5-6.5 mm	0.1-0.5 mm	0.1-0.2 mm
**Length of leaf sheath**	1-2.5 mm	0.5-1 mm	0.5-1 mm	0.5-2 mm	1.5-2 mm
**Leaf arrangement**	Opposite	Whorled-opposite	Opposite-whorled	Opposite-whorled	Opposite
**Number of whorls of leaves**	2	2-3	2-(3)	2-3	2
**Presence of trichomes on leaf margin**	Present	Present	Present	Absent	Absent
**Trichomes density on the leaf margin**	Sparse	Dense	Dense	-	-
**Trichomes type on the leaf margin**	Unicellular-uniseriate	Unicellular-uniseriate	Unicellular-uniseriate	-	-
**The presence of trichomes on the sheath margin**	Present	Present	Present	Absent	Absent
**Trichomes density on sheath margin**	Rare	Sparse	Moderate	-	-
**Trichomes type on the sheath margin**	Unicellular-uniseriate	Unicellular-uniseriate	Unicellular-uniseriate	-	-
**Cone bract of male strobilus**					
**Cone bract size Lx W mm**	2.5-3.2 × 1.2-1.5 mm	1-3 × 0.8-1.2 mm	1.5-2.5 × 0.9-1.5 mm	1.5-2 × 0.9-1 mm	1.5-2 × 0.8-1 mm
**Number of strobili /cone**	7-12	7-10	7-10	7-8	4-6
**Number of pairs of bracts/ cone**	11-17	11-15	11-15	11-12	6-9
**Bract connection**	Free	Fused	Fused	Fused	Fused
**Shape**	Elliptical-obovoid	Oblance-ovate	Elliptical	Elliptical	Narrowly oblong
**Apex**	Acute-rounded	Rounded- obtuse	Obtuse	Rounded	Rounded
**Margin**	Entire	Entire	Ciliate	Entire	Entire
**Length of cone bract: cone**	Less than 1/3	Less than 1/3	Less than 1/3	Less than 1/3	Less than 1/3
**The shape of two bracts (equal or sub-equal)**	Sub equal	Sub equal	Sub equal	Sub equal	Sub equal
**Shape of mid-region**	The whole bract is dark with transparent margin	Very dark middle line and the rest is transparent	Raphe (U- shape)	Raphe (U- shape)	The whole bract is dark with transparent margin

**Trichomes outside**	Glabrous	Hairy	Hairy	Glabrous	Glabrous
**Trichomes density outside**	-	Densely	Densely	-	-
**Trichomes inside**	Hairy	Hairy	Hairy	Glabrous	Glabrous
**Trichomes density inside**	Sparse	Dense	Dense	-	-
**Trichomes type**	Unicellular-uniseriate	Unicellular-uniseriate	Unicellular-uniseriate	-	-
**Male cones**					
**Shape**	Obovate	Oblance-ovate	Oblance-ovate	Elliptical	Orbicular-circular
**Apex**	Retuse-rounded	Rounded-obtuse	Obtuse	Obtuse	Rounded
**Margin**	Entire	Entire	Entire	Entire	Entire
**Number of pairs of inflorescence bracts/ cone**	6-12	7-10	7-8	7-8	4-6
**Base connection**	Free	Fused	Fused	Fused	Fused
**Size of strobilus bract/cone**	1.5-3 × 1-2 mm	1-3 × 0.5-1.5 mm	1.5-2.5 × 0.5-1.5 mm	2.5-3 × 1-1.5 mm	1.5-2 × 1.5-2 mm
**The shape (equal or sub-equal)**	Sub equal	Equal	Equal	Sub equal	Sub equal
**Shape of mid-region**	The whole bract is dark with transparent margin	Have two dark stripes in the middle and transparent margin	The whole bract is dark it makes a raphe at the center and transparent margin	Raphe (U-shape) in the middle and transparent margin	The whole bract is dark with transparent margin
**Trichomes outside**	Hairy	Hairy	Hairy	Glabrous	Glabrous
**Trichomes density outside**	Sparse	Rare	Dense	-	-
**Trichomes inside**	Hairy	Hairy	Hairy	Glabrous	Glabrous
**Trichomes density inside**	Rare	Rare	Dense	-	-
**Trichomes type**	Unicellular-uniseriate	Unicellular-uniseriate	Unicellular-uniseriate	-	-
**The cone looks like (cylindrical or flattened)**	Cylindrical	Cylindrical	Cylindrical	Flattened	Flattened
**Origin of male cone clusters**	Dense axillary	Dense axillary	Terminal	Axillary	Axillary clusters
**Peduncle shape**	Straight	Curved	Straight	Curved	Short straight
**Color**	Yellowish-green	Yellowish-green	Yellowish-green	Yellowish-green	Yellowish-green
**Base**	Obtuse-rounded	Obtuse	Acute-obtuse	obtuse	Rounded

**Number of male cones (bracts)**	6-12	7-10	7-8	7-8	4-6
**Arrangement of strobili**	Opposite	Alternate	Pseudo-opposite	Opposite	Opposite
**The strobili look like (stalked or sessile)**	Stalked	Stalked	Stalked	Sessile	Sessile
**Arrangement of anthers**	Umbellate form	Clustered	Opposite	Clustered	Whorled
**Length of stamens mm**	3-5 mm	2.5-6 mm	2-2.7 mm	3-4.5 mm	2.8-3 mm
**Length of antherophore mm**	2.5-5 mm	2-5.5 mm	1.5-2.5 mm	2.5-4 mm	2-2.5 mm
**Length of floral bract to stamen**	Less than 1/3	Less than 1/3	Nearly equal 1/3	Less than 1/3	Less than 1/3
**The shape of the antherophore column**	Branched above	Un branched above	Un branched above	Un branched above	Un branched above
**Anthers (sessile or stalked)**	Stalked	Sessile	Sessile	Sessile	Sessile
**Number of anthers per strobilus**	3-6	3-4	3	4-7	6-8
**Texture of ripe cones**	Non fleshy with broad scarious margins	Fleshy	Fleshy	Fleshy	Fleshy

Note: - Trichomes density: Rare= 1-2, sparse= 2-3, moderate= 5-6, dense= 6-10 trichomes.

Table S2: Estimating the variations of the percentages (%) of the fatty acids in *Ephedra* species to the gender level using GC-Mass.

Form		Butyric (C4:0)	Valeric (C5:0)	Caproic (C6:0)	Pelargonic (C9:0)	Capric (C10:0)	Undecylic (C11:0)	Lauric (C12:0)	Tridecylic (C13:0)	Myristic (C14:0)	Penta-decylic (C15:0)	Palmitic (C16:0)	Margaric (C17:0)	Stearic (C18:0)	Arachidic (C20:0)	Heneicosylic (C21:0)	Behenic (C22:0)	Lignoceric (C24:0)	Melissic (C30:0)	Sum of saturated FA ± SE
**Saturated fatty acids**																				
		Mean ± SE	Mean ± SE	Mean ± SE	Mean ± SE	Mean ± SE	Mean ± SE	Mean ± SE	Mean ± SE	Mean ± SE	Mean ± SE	Mean ± SE	Mean ± SE	Mean ± SE	Mean ± SE	Mean ± SE	Mean ± SE	Mean ± SE	Mean ± SE	
1	♀	0.0 ± 0.0 a	5.07 ± 0.02 c	0.0 ± 0.0 a	0.0 ± 0.0 a	0.0 ± 0.0 a	0.93 ± 0.14 b	1.01 ± 0.11 b	0.0 ± 0.0 a	2.31 ± 0.02 **c**	1.68 ± 0.11 **d**	16.94 ± 0.87 **a**	2 ± 0.02 **d**	2 ± 0.02 **c**	0.0 ± 0.0 **a**	0.0 ± 0.0 **a**	0.05 ± 0.001 **b**	0.0 ± 0.0 **a**	0.0 ± 0.0 **a**	31.99 ± 2.28 **a**
	♂	//	0.0 ± 0.0 a	0.4 ± 0.01 b	0.20 ± 0.05 c	//	3.25 ± 0.18 **e**	4.17 ± 0.15 e	//	4.44 ± 0.86 **f**	0.0 ± 0.0 **a**	23.19 ± 0.91 **d**	0.0 ± 0.0 **a**	1.58 ± 0.05 **c**	//	//	0.0 ± 0.0 **a**	//	//	37.23 ± 1.13 **b**
2	♀	//	4.25 ± 0.02 b	2.43 ± 0.03 c	0.0 ± 0.0 a	//	3.55 ± 0.05 **f**	4.35 ± 0.07 f	0.68 ± 0.85 **b**	4.65 ± 0.19 **f**	1.91 ± 0.04 **e**	29.60 ± 2.31 **f**	//	3.60 ± 0.01 **e**	//	//	//	//	//	55.02 ± 1.43 **f**
	♂	//	0.0 ± 0.0 a	0.0 ± 0.0 a	//	0.18 ± 0.02 c	1.47 ± 0.04 **d**	2.95 ± 0.39 d	0.80 ± 0.08 c	3.44 ± 0.01 **d**	1.97 ± 0.21 **f**	28.64 ± 2.90 **e**	2.56 ± 0.07 **e**	5.34 ± 0.15 **h**	0.1 ± 0.002 **b**	0.15 ± 0.06 **b**	0.1 ± 0.002 **c**	0.17 ± 0.02 **b**	//	47.87 ± 1.49 **d**
3	♀	//	//	//	0.12 ± 0.03 b	0.10 ± 0.05 b	1.21 ± 0.09 **c**	1.25 ± 0.90 c	0.0 ± 0.0 a	14.23 ± 0.51 **g**	12.02 ± 0.34 **g**	33.20 ± 0.44 **h**	0.96 ± 0.05 **c**	0.96 ± 0.05 **b**	0.0 ± 0.0 **a**	0.0 ± 0.0 **a**	0.0 ± 0.0 **a**	0.0 ± 0.0 **a**	//	64.05 ± 2.12 **h**
	♂	17.35 ± 0.04 c	//	//	0.0 ± 0.0 a	0.0 ± 0.0 a	0.0 ± 0.0 a	0.0 ± 0.0 a	//	27.25 ± 0.46 **h**	0.0 ± 0.0 **a**	22.23 ± 1.03 **c**	3.98 ± 0.11 **h**	3.96 ± 0.13 **f**	//	//	5.13 ± 0.02 **f**	//	//	79.9 ± 3.18 **j**
4	♀	0.0 ± 0.0 a	//	//	//	//	//	//	//	3.62 ± 0.06 **e**	14.19 ± 0.04 **h**	41.32 ± 1.46 **i**	0.69 ± 0.03 **b**	15.15 ± 1.12 **i**	0.49 ± 0.02 **c**	//	0.0 ± 0.0 **a**	//	//	75.46 ± 3.27 **i**
	♂	5.63 ± 0.05 b	//	//	//	//	//	//	//	1.68 ± 0.07 **b**	1.52 ± 0.17 **b**	31.65 ± 1.02 **g**	3.11 ± 0.09 **f**	3.11 ± 0.08 **d**	0.0 ± 0.0 **a**	//	0.72 ± 0.09 **e**	//	1.39 ± 0.05 **b**	48.81 ± 2.26 **e**
5	♀	0.0 ± 0.0 a	//	//	//	//	//	//	//	0.0 ± 0.0 **a**	1.61 ± 0.01 **c**	18.73 ± 1.20 **b**	4.24 ± 0.04 **i**	4.24 ± 0.08 **g**	//	//	0.41 ± 0.06 **d**	//	0.0 ± 0.0 **a**	40.93 ± 1.37 **c**
	♂	//	//	//	//	//	//	//	//	//	0.0 ± 0.0 **a**	55.79 ± 0.38 **j**	3.48 ± 0.06 **g**	0.0 ± 0.0 **a**	//	//	0.0 ± 0.0 **a**	//	//	59.27 ± 2.42 **g**

Form		Myristoleic (C14:1 cis 9)	14-Pentadecenoic (C15:1)	Palmitoleic (C16:1 cis 9)	11-Hexadecenoic (C16:1 cis 11)	10-Heptadecenoic (C17:1 cis 10)	Oleic (C18:1 cis 9)	Elaidic (C18:1 trans 9)	cis-10-oleic (C18:1 cis 10)	Vaccenic (C18:1 cis 11)	13-Octadecenoic (C18:1 cis 13)	16-Octadecenoic (C18:1 trans 16)	17-Octadecenoic (C18:1)	10-Nonadecenoic (C19:1 cis 10)	13-Nonadecenoic (C19:1 cis 13)	Gondoic (C20:1 cis 11)	Paullinic (C20:1 cis 13)	Sum of monounsaturated FA ± SE
		Mean ± SE	Mean ± SE	Mean ± SE	Mean ± SE	Mean ± SE	Mean ± SE	Mean ± SE	Mean ± SE	Mean ± SE	Mean ± SE	Mean ± SE	Mean ± SE	Mean ± SE	Mean ± SE	Mean ± SE	Mean ± SE	
1	♀	0.0 ± 0.0 a	0.0 ± 0.0 **a**	1.95 ± 0.01 **d**	0.0 ± 0.0 **a**	0.0 ± 0.0 **a**	3.2 ± 0.05 **c**	0.0 ± 0.0 **a**	0.0 ± 0.0 **a**	0.0 ± 0.0 **a**	0.0 ± 0.0 **a**	0.0 ± 0.0 **a**	0.0 ± 0.0 **a**	0.0 ± 0.0 **a**	0.0 ± 0.0 **a**	0.0 ± 0.0 **a**	0.0 ± 0.0 **a**	5.15 ± 2.02 a
	♂	//	//	7.70 ± 0.08 **i**	//	//	13.20 ± 0.18 **f**	8.15 ± 0.12 **e**	//	10.13 ± 0.13 **f**	9.12 ± 0.30 **g**	//	//	//	//	//	//	48.30 ± 2.37 h
2	♀	//	//	5.76 ± 0.003 **h**	//	//	11.43 ± 0.22 **e**	8.50 ± 0.11 **e**	//	0.0 ± 0.0 **a**	0.0 ± 0.0 **a**	//	//	//	//	//	//	25.69 ± 1.15 d
	♂	//	//	4.66 ± 0.14 **e**	//	1.24 ± 0.01 **b**	22.01 ± 0.38 **h**	1.02 ± 0.02 **b**	//	1.04 ± 0.05 **b**	0.94 ± 0.10 **c**	//	//	0.15 ± 0.01 **b**	0.13 ± 0.001 **b**	0.1 ± 0.001 **b**	0.09 ± 0.002 **b**	31.38 ± 1.12 f
3	♀	0.08 ± 0.01 b	0.26 ± 0.03 **b**	5.68 ± 0.21 **g**	//	0.0 ± 0.0 **a**	11.87 ± 0.19 **e**	4.78 ± 0.09 **c**	//	5.19 ± 0.09 **c**	1.89 ± 0.20 **d**	//	//	0.0 ± 0.0 **a**	0.0 ± 0.0 **a**	0.0 ± 0.0 **a**	0.09 ± 0.003 **b**	29.84 ± 0.06 e
	♂	0.0 ± 0.0 a	0.0 ± 0.0 **a**	2.60 ± 0.07 **f**	//	//	0.0 ± 0.0 **a**	0.0 ± 0.0 **a**	8.94 ± 0.03 **b**	0.0 ± 0.0 **a**	0.0 ± 0.0 **a**	8.56 ± 0.80 **c**	//	//	//	//	0.0 ± 0.0 **a**	20.1 ± 0.08 b
4	♀	//	//	1.20 ± 0.03 **b**	1.02 ± 0.01 **b**	//	5.98 ± 0.09 **d**	5.09 ± 0.06 **d**	0.0 ± 0.0 **a**	6.87 ± 0.11 **d**	0.06 ± 0.01 **b**	0.0 ± 0.0 **a**	//	//	//	//	//	20.22 ± 1.12 b
	♂	//	//	5.47 ± 0.08 **g**	0.0 ± 0.0 **a**	//	19.48 ± 0.35 **g**	9.70 ± 0.01 **f**	//	9.78 ± 0.18 **e**	5.60 ± 0.08 **f**	//	//	//	//	//	//	50.03 ± 1.55 i
5	♀	//	//	0.0 ± 0.0 **a**	//	//	1.89 ± 0.03 **b**	0.0 ± 0.0 **a**	± 11.27 0.07**d**	0.0 ± 0.0 **a**	0.0 ± 0.0 **a**	11.07 ± 0.18 **d**	//	//	//	//	//	24.23 ± 1.18 c
	♂	//	//	1.36 ± 0.02 **c**	//	//	0.0 ± 0.0 **a**	8.01 ± 0.01 **e**	9.11 ± 0.03 **c**	13.73 ± 0.22 **g**	3.75 ± 0.02 **e**	1.31 ± 0.06 **b**	3.46 ± 0.04 **b**	//	//	//	//	40.73 ± 1.45 g

Form		Polyunsaturated fatty acids																	
		7,10-Hexadecadienoic (Z,Z) (C16:2)	9,12-Hexadecadienoic (Z,Z) (C16:2)	Linoleic (C18:2)	Rumenic (C18:2)	8,11-Octadecadienoic (E,E) (C18:2)	6,9-Octadecadienoic (E,E) (C18:2)	Linoelaidic (C18:2)	α-Linolenic (C18:3)	γ-linolenic (C18:3)	8,11-Icosadienoic (C20:2)	11,14-Icosadienoic (C20:2)	Dihomo-γ-linolenic (C20:3)	7,10,13-Eicosatrienoic (C20:3)	Arachidonic (C20:4)	Timnodonic (EPA) (C20:5)	6,9,12,15-Docosatetraenoic (C22:4)	Cervonic (C22:6)	Sum of polyunsaturated FA ± SE
		Mean ±SE	Mean ± SE	Mean ± SE	Mean ± SE	Mean ± SE	Mean ± SE	Mean ± SE	Mean ± SE	Mean ± SE	Mean ± SE	Mean ± SE	Mean ± SE	Mean ± SE	Mean ± SE	Mean ± SE	Mean ± SE	Mean ± SE	
1	♀	7.62 ± 0.07 d	3.80 ± 0.02 d	26.95 ± 1.86 i	9.29 ± 0.26 d	0.0 ± 0.0 a	0.0 ± 0.0 a	0.0 ± 0.0 a	5.36 ± 0.01 c	7.82 ± 0.04 c	1.15 ± 0.05 b	0.0 ± 0.0 a	0.0 ± 0.0 a	0.0 ± 0.0 a	0.16 ± 0.01 b	0.19 ± 0.08 b	0.16 ± 0.01 b	0.36 ± 0.03 b	62.86 ± 2.51 i
	♂	0.78 ± 0.01 b	0.72 ± 0.02 b	6.16 ± 0.25 e	0.0 ± 0.0 a	3.14 ± 0.03 e	//	//	0.75 ± 0.03 b	0.0 ± 0.0 a	0.0 ± 0.0 a	1.17 ± 0.07 b	1.75 ± 0.05 b	//	0.0 ± 0.0 a	0.0 ± 0.0 a	0.0 ± 0.0 a	0.0 ± 0.0 a	14.47 ± 1.16 e
2	♀	4.73 ± 0.02 c	2.71 ± 0.002 c	11.85 ± 0.17 g	//	0.0 ± 0.0 a	//	//	0.0 ± 0.0 a	//	//	0.0 ± 0.0 a	0.0 ± 0.0 a	//	//	//	//	//	19.29 ± 1.11 f
	♂	0.0 ± 0.0 a	0.0 ± 0.0 a	10.17 ± 0.21 f	2.02 ± 0.05 c	//	//	//	5.52 ± 0.01 c	2.92 ± 0.03 b	//	//	//	0.12 ± 0.01 b	//	//	//	//	20.75 ± 0.98 g
3	♀	//	//	2.04 ± 0.01 d	1.88 ± 0.04 b	1.91 ± 0.01 d	0.28 ± 0.01 b	//	0.0 ± 0.0 a	0.0 ± 0.0 a	//	//	//	0.0 ± 0.0 a	//	//	//	//	6.11 ± 0.23 d
	♂	//	//	0.0 ± 0.0 a	0.0 ± 0.0 a	0.0 ± 0.0 a	0.0 ± 0.0 a	//	//	//	//	//	//	//	//	//	//	//	0.0 ± 0.0 a
4	♀	//	//	1.09 ± 0.01 b	//	0.49 ± 0.02 c	1.04 ± 0.05 c	1.12 ± 0.02 b	//	//	//	//	//	//	//	//	//	//	4.32 ± 0.09 c
	♂	//	//	1.16 ± 0.01 c	//	0.0 ± 0.0 a	0.0 ± 0.0 a	0.0 ± 0.0 a	//	//	//	//	//	//	//	//	//	//	1.16 ± 0.03 b
5	♀	//	//	18.26 ± 0.32 h	16.26 ± 0.29 e	0.32 ± 0.01 b	//	//	//	//	//	//	//	//	//	11.70 ± 0.32 c	//	//	34.84 ± 1.29 h
	♂	//	//	0.0 ± 0.0 a	0.0 ± 0.0 a	0.0 ± 0.0 a	//	//	//	//	//	//	//	//	//	0.0 ± 0.0 a	//	//	0.0± 0.0 a

Note*:* **1**.*E. alata;* **2**. *E. aphylla;* **3**. *E.ciliata;* **4**. *E. foemina;* **5**. *E. pachyclada*
♀*: female;* ♂: male; //: 0.0 ± 0.0 a
